# Genetic counselling of a male patient with hypohidrotic ectodermal dysplasia

**DOI:** 10.1186/1746-160X-8-S1-P4

**Published:** 2012-05-25

**Authors:** G Kovács, E Endreffy, Z Maróti

**Affiliations:** 1 Department of Pediatrics, University of Szeged, Hungary

## 

Hypohidrotic ectodermal dysplasias (HED) are characterized by abnormal morphogenesis of epidermis and epidermal appendages. They may be inherited in an autosomal dominant, autosomal recessive or X-linked recessive manner. The most common type shows X-linked inheritance, and males.are usually more severely affected than females. In a male infant who was treated in our department, the diagnosis of HED was made 28 years ago. His mother and grandmother also show typical signs of HED in a milder form, while the other family members are all healthy. This young man wanted to know his risk of having affected children. We examined the segregation of X-chromosomes in ten members of the family with 2 intragenic and one extragenic short tandem repeat (STR) markers of the gene *EDA* (Xq12-13). According to our results, only family members with clinical signs of HED had the same X-chromosome (the affected son, his mother and grandmother), but none of the healthy family members. Direct mutation analysis of *EDA* was performed, but no aberration could be detected in this gene. Nevertheless, genetic counselling was possible based on the results of the segregation analysis. In this situation, the male progeny of the patient will not be affected, while female progeny will be carriers.

